# Predicting a clinically important outcome in patients with low back pain following McKenzie therapy or spinal manipulation: a stratified analysis in a randomized controlled trial

**DOI:** 10.1186/s12891-015-0526-1

**Published:** 2015-04-01

**Authors:** Tom Petersen, Robin Christensen, Carsten Juhl

**Affiliations:** Back Center Copenhagen, Copenhagen, Denmark; Department of Rheumatology, Musculoskeletal Statistics Unit, The Parker Institute, Copenhagen University Hospital, Frederiksberg, Denmark; Department of Sports Science and Clinical Biomechanics, Research Unit for Musculoskeletal Function and Physiotherapy, University of Southern Denmark, Odense, Denmark; Department of Orthopedics, University Hospital of Copenhagen, Gentofte, Denmark

**Keywords:** Low back pain, McKenzie, Spinal manipulation, Predictive value, Effect modification

## Abstract

**Background:**

Reports vary considerably concerning characteristics of patients who will respond to mobilizing exercises or manipulation. The objective of this prospective cohort study was to identify characteristics of patients with a changeable lumbar condition, i.e. presenting with centralization or peripheralization, that were likely to benefit the most from either the McKenzie method or spinal manipulation.

**Methods:**

350 patients with chronic low back pain were randomized to either the McKenzie method or manipulation. The possible effect modifiers were age, severity of leg pain, pain-distribution, nerve root involvement, duration of symptoms, and centralization of symptoms. The primary outcome was the number of patients reporting success at two months follow-up. The values of the dichotomized predictors were tested according to the prespecified analysis plan.

**Results:**

No predictors were found to produce a statistically significant interaction effect. The McKenzie method was superior to manipulation across all subgroups, thus the probability of success was consistently in favor of this treatment independent of predictor observed. When the two strongest predictors, nerve root involvement and peripheralization, were combined, the chance of success was relative risk 10.5 (95% CI 0.71-155.43) for the McKenzie method and 1.23 (95% CI 1.03-1.46) for manipulation (P = 0.11 for interaction effect).

**Conclusions:**

We did not find any baseline variables which were statistically significant effect modifiers in predicting different response to either McKenzie treatment or spinal manipulation when compared to each other. However, we did identify nerve root involvement and peripheralization to produce differences in response to McKenzie treatment compared to manipulation that appear to be clinically important. These findings need testing in larger studies.

**Trial registration:**

Clinicaltrials.gov: NCT00939107

**Electronic supplementary material:**

The online version of this article (doi:10.1186/s12891-015-0526-1) contains supplementary material, which is available to authorized users.

## Background

The most recent published guidelines for the treatment of patients with persistent non-specific low back pain (NSLBP) recommend a program focusing on self-management after initial advice and information. These patients should also be offered structured exercises tailored to the individual patient and other modalities such as spinal manipulation [1,2].

Previous studies have compared the effect of the McKenzie-method, also known as Mechanical Diagnosis and Therapy (MDT), with that of spinal manipulation (SM) in heterogeneous populations of patients with acute and subacute NSLBP and found no difference in outcome [3,4].

Recently, the need for studies testing the effect of treatment strategies for subgroups of patients with NSLBP in primary care has been emphasized in consensus-papers [5,6] as well as the current European guidelines [7], based on the hypothesis that subgroup analyses, preferably complying with the recommendations of “Prognostic Factor Research”[8], will improve decision making towards the most effective management strategies. Although initial data show promising results, there is presently insufficient evidence to recommend specific methods of subgrouping in primary care [1,9].

Three randomized studies, comprising patients with predominantly acute or subacute low back pain (LBP), have tested the effects of MDT versus SM in a subgroup of patients that presented with centralization of symptoms or directional preference (favorable response to end range motions) during physical examination [10-12]. The conclusions drawn from these studies were not in concurrence and the usefulness was limited by a low methodological quality.

Our recent randomized study, comprising patients with predominantly chronic LBP (CLBP), found a marginally better overall effect of MDT versus SM in an equivalent group [13]. In order to pursue the idea of subgrouping further, it was part of the study plan to explore predictors based on patient characteristics that could assist the clinician in targeting the most favorable treatment to the individual patient.

The objective of this study was to identify subgroups of patients with predominantly CLBP, presenting with centralization or peripheralization, which were likely to benefit from either MDT or SM two months after the completion of treatment.

## Methods

### Data collection

The present study is a secondary analysis of a previously published randomized controlled trial [13]. We recruited 350 patients from September 2003 through May 2007 at an outpatient back care centre in Copenhagen, Denmark.

### Patients

Patients were referred from primary care physicians for treatment of persistent LBP. Eligible patients were between 18 and 60 years of age, suffering from LBP with or without leg pain for a period of more than 6 weeks, able to speak and understand the Danish language, and fulfilled the clinical criteria for centralization or peripheralization of symptoms during initial screening. Centralization was defined as the abolition of symptoms in the most distal body region (such as the foot, lower leg, upper leg, buttocks, or lateral low back) and peripheralization was defined as the production of symptoms in a more distal body region. These findings have previously been found to have acceptable degree of inter-tester reliability (Kappa value 0.64) [14]. The initial screening was performed prior to randomization by a physical therapist with a diploma in the MDT examination system. Patients were excluded if they were free of symptoms at the day of inclusion, demonstrated positive non-organic signs [15], or if serious pathology, i.e. severe nerve root involvement (disabling back or leg pain in combination with progressive disturbances in sensibility, muscle strength, or reflexes), osteoporosis, severe spondylolisthesis, fracture, inflammatory arthritis, cancer, or referred pain from the viscera, was suspected based on physical examination and/or magnetic resonance imaging. Other exclusion criteria were application for disability pension, pending litigation, pregnancy, co-morbidity, recent back surgery, language problems, or problems with communication including abuse of drugs or alcohol.

The trial population had predominantly CLBP lasting on average 95 weeks (SD 207), mean age was 37 years (SD10), mean level of back and leg pain was 30 (SD 11.9) on a Numeric Rating Scale ranging from 0 to 60, and mean level of disability was 13 (SD 4.8) on Roland Morris Disability Questionnaire (0-23). Our method of pain measurement reflects that back pain is often a fluctuating condition where pain location and severity might vary on a daily basis. Therefore, a validated comprehensive pain questionnaire [16] was used in order to guarantee that all aspects of back and leg pain intensity were recorded. The scales are outlined in the legend to Table 1.

**Table 1:** **Comparison of distribution of baseline variables between groups**

	**McKenzie group**			**Manipulation group**				
	**Number of patients**	**%**	**Total**	**Number of patients**	**%**	**Total**	**RR (95% CI)**	**P-value**
Age								
Below 40	107	61.6%	175	117	66.9%	175	0.91 (0.78-1.07)	0.27
Gender								
Male	72	41.1%	175	83	47.4%	175	0.87 (0.69-1.10)	0.24
Duration of symptoms								
More than a year	55	31.4%	175	54	30.9%	175	1.01 (0.88-1.16)	0.91
Disability								
Mild/moderate¤	64	36.6%	175	68	38.9%	175	0.94 (0.72-1.23)	0.66
Leg pain¤¤								
Moderate/severe	92	52.6%	175	87	49.7%	175	1.06 (0.85-1.32)	0.59
Back pain¤¤								
Mild	19	10.9%	175	24	13.7%	175	0.79 (0.45-1.39)	0.42
Sickleave past year								
Six days or less¤¤¤	85	52.1%	163	87	52.7%	165	0.99 (0.80-1.20)	0.92
Nerve root involvement								
Yes*	18	10.3%	175	16	9.1%	175	1.13 (0.59-3.13)	0.72
Pain below the knee								
Yes	88	50.3%	175	102	58.3%	175	0.86 (0.71-1.05)	0.13
Expectations to recovery								
High**	84	52.5%	160	68	49.3%	138	1.07 (0.85-1.33)	0.58
Expectations to work								
High***	63	37.1%	170	76	43.9%	173	0.84 (0.65-1.09)	0.20
Pain response								
Centralization§	151	86.3%	175	156	89.1%	175	0.97 (0.89-1.05)	0.42

N = 350 except Sick leave past year (N = 328), Expectations to recovery (N = 298) and Expectations to work (N = 343).

RR = Relative Risks (95% confidence intervals) show the chance of having the characteristics in the McKenzie group compared to the Manipulation group (i.e. the chance of having pain below the knee in the McKenzie group compared to the Manipulation group).

¤The total score on Roland Morris were divided into mild (0-5), moderate (6-11), or severe (12-23) disability.

¤¤Each of the back and leg pain questionnaires included 3 separate 11 point box scales (0-30) comprising the following items: pain at the moment, the worst pain within the past two weeks, and the average level of pain within the last two weeks. For each questionnaires, these summed to a total score ranging from 0 points (no back or leg pain at all) to 30 points (worst possible back or leg pain). The total score was divided into mild (0-10), moderate (11-20), or severe (21-30) pain.

¤¤¤Number of days reported by the patient. Dichotomized into high/low risk groups according to scores above/below the median of 6 found in the sample.

*Based on presence of dominant leg pain in any distribution as well as the following clinical signs of nerve root pain: positive straight leg raise test of less than 60 degrees that reproduced leg pain in combination with diminished reflexes, and/or muscle weakness in a myotomal or dermatomal pattern, and/or sensory disturbances.

**Scored before the initiation of third treatment on an 11-point box scale. 0 indicates I expect no improvement at all; 10, I am certain that I will improve. Dichotomized into high/low risk groups according to the median scores: high (8 or above), low (below 8).

***Measured on an 11 point box scale regarding expectations about coping with work tasks in six weeks time (endpoints ‘No trouble at all’ and ‘So much trouble that I won’t be able to do my job at all’). Dichotomized into high/low risk groups according to the median scores low (3 or above); high (below 3).

§Movement of symptoms from a distal to a more proximal location during pre-randomization physical screening.

After baseline measures were obtained, randomisation was carried out by a computer-generated list of random numbers in blocks of ten using sealed opaque envelopes.

### Ethics

Ethical approval of the study was granted by Copenhagen Research Ethics Committee, file no 01-057/03. All patients received written information about the study and gave their written consent prior to participation.

### Treatments

The practitioners performing the treatments had no knowledge of the results of the initial screening. The treatment programs were designed to reflect daily practice as much as possible. Detailed information on these programs have been published earlier [13].

The MDT treatment was planned individually following the therapist’s pre-treatment physical assessment. Specific manual vertebral mobilization techniques including high velocity thrust were not allowed. An educational booklet describing self care [17] or a “lumbar roll” for correction of the seated position was sometimes provided to the patient at the discretion of the therapist. In the SM treatment, high velocity thrust was used in combination with other types of manual techniques. The choice of combination of techniques was at the discretion of the chiropractor. General mobilizing exercises, i.e. self-manipulation, alternating lumbar flexion/extension movements, and stretching, were allowed but not specific exercises in the directional preference. An inclined wedged pillow for correction of the seated position was available to the patients if the chiropractor believed this to be indicated.

In both treatment groups, patients were informed thoroughly of the results of the physical assessment, the benign course of back pain, and the importance of remaining physically active. Guidance on proper back care was also given. In addition, all patients were provided with a Danish version of “The Back Book” which previously has been shown to have beneficial effect on patients’ beliefs about back pain [18]. A maximum of 15 treatments for a period of 12 weeks were given. If considered necessary by the treating clinician, patients were educated in an individual program of self-administered mobilizing, stretching, stabilizing, and/or strengthening exercises at the end of the treatment period. Treatments were performed by clinicians with several years of experience. Patients were instructed to continue their individual exercises at home or at a gym for a minimum of two months after completion of the treatment at the back center. Because the patients suffered predominantly from CLBP we expected this period of self administered exercises to be necessary for the patients to experience the full effect of the intervention. Patients were encouraged not to seek any other kind of treatment during this two months period of self-administered exercises.

### Outcome measures

The primary outcome was the proportion of patients reporting success at follow-up two months after end of treatment. Treatment success was defined as a reduction of at least 5 points or a final score below 5 points on the 23-item modified Roland Morris Disability Questionnaire (RMDQ) [19]. A validated Danish version of RMDQ was used [20]. The definition of treatment success was based on the recommendations by others [21,22]. A sensitivity analysis using 30% relative improvement on RMDQ as definition of success was also performed. In accordance with the protocol [13], we considered a relative between-group difference of 15% in the number of patients with successful outcome to be minimal clinically important in our analysis of interaction.

### Prespecified predictor variables

In order to reduce the likelihood of spurious findings [23], we restricted the number of candidate effect modifiers in the dataset to six. To increase the validity of our findings, a directional hypothesis was established for each variable according to the recommendations of Sun et al. [24] Four baseline variables have previously been suggested in randomized studies to be predictive of long term good outcome in patients with persistent LBP following MDT in comparison with strengthening training: centralization [25,26], or following SM in comparison to physiotherapy or treatment chosen by a general practitioner: age below 40 years [27,28], duration of symptoms more than 1 year [27], and pain below the knee [29]. As recommended by others [30], another two variables were added based on the participating experienced clinicians’ judgments of which characteristics they would expect to predict good outcome from their treatment compared to the other. The additional variables prioritized by the physiotherapists in the MDT group were signs of nerve root involvement and substantial leg pain. The additional variables prioritized by the chiropractors in the SM group were no signs of nerve root involvement and not substantial leg pain.

In a supplementary analysis, we took the opportunity to explore whether the inclusion of further six baseline variables, assumed to have prognostic value for good outcome in either of the treatment groups, would appear to have an effect modifying effect as well. To our knowledge, no further variables from previous one arm studies have been reported to have prognostic value of long term good outcome in patients with persistent LBP following MDT, whereas three variables have been reported to have prognostic value following SM: male gender [28], mild disability [28], and mild back pain [28]. Another three variables were agreed upon by the clinicians to be included in the supplementary analysis as they were assumed by experience from clinical practice to have prognostic value for good outcome regardless of treatment with MDT or SM: low number of days on sick leave past year, high patient expectations to recovery, and high patient expectations about coping with work tasks six weeks after initiation of treatment.

Dichotomization of possible predictor variables were made to allow for comparisons to be made with those of earlier studies. In cases where no cut off values could be found in the literature, dichotomization was performed above/below the median found in the sample. Definitions of variables are presented in the legend to Table 1.

### Statistics

The entire intention-to-treat (ITT) population was used in all the analyses. The last score was carried forward for subjects with missing two months RMDQ scores (7 patients in the MDT group and 14 patients in the SM group). In addition, a post hoc per protocol analysis was carried out comprising only those 259 patients that completed the full treatment. The analysis plan was agreed in advance by the trial management group.

The possible predictors were dichotomized and the chance of success was investigated by estimating the relative risk (RR) of success in each of the two strata. The impact of the investigated predictors was estimated by comparing the chance of success between the treatment groups when divided into the two strata. To test for treatment effect modification of the predictors we performed chi-squared tests for interaction between intervention and the two different strata for each of the predictors. This is basically the same as an interaction from a regression model. Confidence intervals were also inspected for potential clinically important effects.

Following the univariate analysis, a multivariate analysis was planned including effect modifiers with a p-value below 0.1.

## Results

Participants were similar with respect to socio-demographic and clinical characteristics at baseline in the treatment groups. An overview of the distribution of the included dichotomized variables at baseline is provided in Table 1. No differences were found between the treatment groups.

Overall, the post hoc per protocol analysis did not produce outcome results that were different from the results of the ITT analysis and therefore only the results of the ITT analysis will be reported.

**Figure 1:**
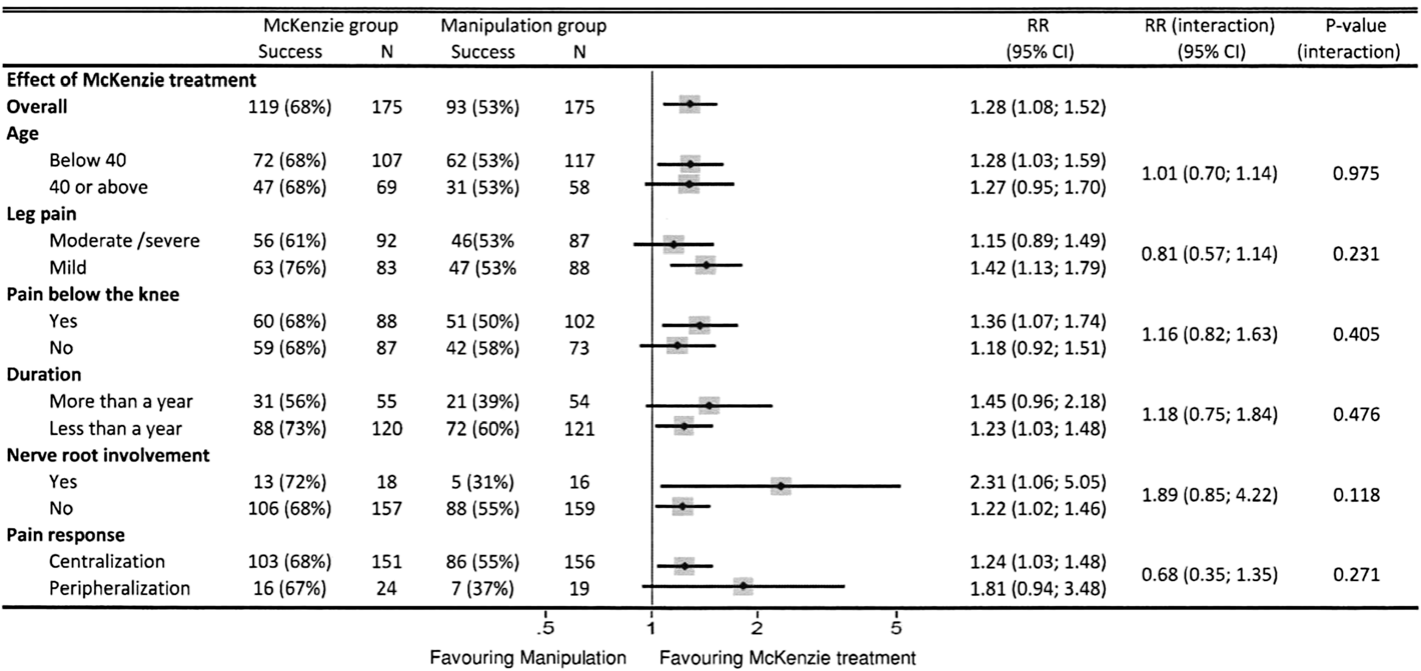
**Treatment effect modified by predictors.** The top point estimate and confidence intervals indicate overall effect without subgrouping. Subsequent pairs of point estimates and confidence intervals show the chances of treatment success following MDT vs spinal manipulation in six subgroups.

Figure 1 presents the distribution of predictors with regards to effect modification in the MDT group versus SM. In all subgroups, the probability of success with MDT was superior to that of SM. Because of low sample size, confidence intervals were wide and none of the predictors had a statistically significant treatment modifying effect. The predictors with a clinically important potential effect in favor of MDT compared to SM were nerve root involvement (28% higher proportion of patients with success when nerve root involvement was present than when absent) and peripheralization of symptoms (17% higher proportion of patients with success in case of peripheralization than in case of centralization). If present, nerve root involvement increased the chance of success following MDT 2.31 times compared to that of SM and 1.22 times if not present. This means that for the subgroup of patients with nerve root involvement receiving MDT, compared to those receiving SM, the relative effect appeared to be 1.89 times (2.31/1.22, P = 0.118) higher than for the subgroup with no nerve root involvement.

**Figure 2:**
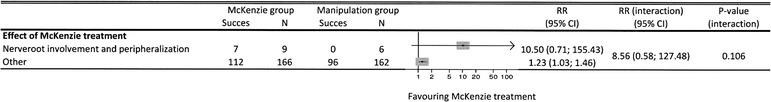
**Impact of the two clinically important predictors combined on treatment effect.** RR = Relative Risk with Yates correction.

Figure 2 presents the modifying effect of a composite of the two predictors with a clinically important potential effect. If signs of nerve root involvement and peripheralization were present at baseline, the chance of success with MDT compared to SM appeared 8.5 times higher than for the subgroup with no centralization and nerve root involvement. The number of patients was very small and the differences were not statistically significant (P = 0.11).

None of the prognostic candidate variables explored in the supplementary analysis appeared to have any clinically important modifying effect (Additional file 1: Table S1).

The results from the sensitivity analysis using 30% relative improvement on RMDQ as definition of success were not markedly different from those presented above (Additional file 2: Table S2).

## Discussion

To our knowledge, this is the first study trying to identify effect modifiers when two mobilizing strategies, i.e. MDT and SM, are compared in a sample of patients with as changeable condition characterized by centralization or peripheralization.

Our study found that none of the potential effect modifiers were able to statistically significantly increase the overall effect of MDT compared to that of SM. However, the between-group difference for two of the variables exceeded our clinically important success-rate of 15% in number of patients with successful outcome, so our study is likely to have missed a true effect and, in that sense, did not have a large enough sample size.

The most apparent finding is that in our small subgroup of patients with signs of nerve root involvement, the relative chance of success appeared 1.89 times (2.31/1.22) higher than in patients with no nerve root involvement when treated with MDT, compared to those treated with SM. The difference was in the expected direction.

Although not statistically significant in our small sample, the variable peripheralization exceeded our clinically important success-rate of 15%, but was found not to be in the expected direction. No previous studies have assessed the effect modification of centralization or peripheralization in patients with CLBP. The RCT by Long et al. [25,26] concluded that patients with directional preference, including centralization, fared better 2 weeks after baseline than patients with no directional preference when treated with MDT in comparison with strengthening training. However, the outcome among peripheralizers was not reported, so the poor outcome reported in patients with no directional preference might be related to the subgroup of patients who responded with no change in symptoms during initial examination and not to those that responded with peripheralization. An alternative explanation might be that the effect modifying impact of centralization or peripheralization on MDT is dependent on the control treatment. Our findings suggest that future studies in this area need to involve predictive value of peripheralization as well as centralization.

When a composite of the two most promising predictors, peripheralization and signs of nerve root involvement, were present at baseline, the relative chance of success with MDT compared to SM appeared 8.5 times higher than for the subgroup with no centralization and nerve root involvement. The number of patients was very small and the confidence interval was wide. Therefore only a preliminary conclusion about interaction can be drawn and it calls for a validation in future studies.

In our study, there appeared to be no characteristic by which SM had better results compared to MDT. Thus, we could not support the results of two studies with a similar design as ours (two arms, sample of patients with persistent LBP, and outcome reported in terms of reduction of disability at long term follow up) [27,29]. In those studies, Nyiendo et al. [29] found a modifying effect of leg pain below knee on treatment by SM compared to that of the general practitioner six months after baseline, and Koes et al. [27] found a modifying effect of age below 40 years and symptom duration more than a year on treatment by SM compared to that of physiotherapy 12 months after baseline. However, results from those, as well as other previous RCTs comprising patients with persistent LBP, have supported our findings regarding the lack of effect modification of age [27,29,31], sex [29,31], baseline disability [27,29,31], and duration of symptoms [31], on SM when measured on reduction of disability 6-12 months after randomization. So, although evidence is emerging in patients with acute LBP regarding subgroup characteristics predictive of better results from SM compared to other types of treatment [32], we are still in the dark with respect to patients with persistent LBP.

The usefulness of choosing a criterion for success by combining an improvement of at least 5 points or an absolute score below 5 points on RMDQ is debatable. A total of 22 patients were considered successful based on score below 5 at follow up without having an improvement of at least 5 points. We therefore performed a sensitivity analysis using a relative improvement of at least 30% as criterion of success as recommended by others [22] (see Additional file 2: Table S2). As a result, the percentage of patients with successful outcome in the MDT group remained the same whereas 4 more patients were defined as successes in the SM group. Overall the sensitivity analysis did not produce outcome results that were markedly different from those of the primary analysis and therefore only those have been discussed above.

### Strengths and limitations

This study used data from a RCT, whereas many others have used single arm designs not suitable for the purpose of evaluating treatment effect modification [33]. In accordance with the recommendations by the PROGRESS group [8] we prespecified the possible predictors and also the direction of the effect. Furthermore, we limited the number of predictors included in order to minimize the chance of spurious findings.

The main limitation in secondary studies to previously conducted RCTs is that they are powered to detect overall treatment effect rather that effect modification. In recognition of the post hoc nature of our analysis, reflected in wide confidence intervals, we must emphasize that our findings are exploratory and require formal testing in a larger sample size.

## Conclusions

In all subgroups, the probability of success with MDT was superior to that of SM. Although not statistically significant, the presence of nerve root involvement and peripheralization appear promising effect modifiers in favour of MDT. These findings need testing in larger studies.

## Additional files

Additional file 1: Table S1. Treatment effect modified by prognostic variables. Additional file 2: Table S2. Results of the sensitivity analysis. Treatment effect modified by predictors when 30% relative improvement on RMDQ as definition of success was used.
