# Supplementary material for: Predicting a clinically important outcome in patients with low back pain following McKenzie therapy or spinal manipulation: a stratified analysis in a randomized controlled trial
Source: BMC Musculoskelet Disord. 2015 Apr 1;16:74. doi: 10.1186/s12891-015-0526-1 (PMC4393582; doi:10.1186/s12891-015-0526-1)
Supplement: Additional file 1: Table S1. — Treatment effect modified by prognostic variables. [file 12891_2015_526_MOESM1_ESM.docx]

**Appendix file 3 Table S3**. Treatment effect modified by prognostic variables.
