# Supplementary material for: Predicting a clinically important outcome in patients with low back pain following McKenzie therapy or spinal manipulation: a stratified analysis in a randomized controlled trial
Source: BMC Musculoskelet Disord. 2015 Apr 1;16:74. doi: 10.1186/s12891-015-0526-1 (PMC4393582; doi:10.1186/s12891-015-0526-1)
Supplement: Additional file 2: Table S2. — Results of the sensitivity analysis. Treatment effect modified by predictors when 30% relative improvement on RMDQ as definition of success was used. [file 12891_2015_526_MOESM2_ESM.docx]

**Additional file 4 Table S4**. Results of the sensitivity analysis. Treatment effect modified by predictors when 30% relative improvement on RMDQ as definition of success was used.
